# Papillary Fibroelastoma of the Right Ventricular Free Wall

**DOI:** 10.1155/2014/654641

**Published:** 2014-09-02

**Authors:** Tetsuya Niino, Satoshi Unosawa

**Affiliations:** Department of Cardiovascular Surgery, National Hospital Organization Disaster Medical Center, 3256 Midoricho, Tachikawa, Tokyo 190-0014, Japan

## Abstract

Papillary fibroelastoma is a rare benign cardiac tumor that usually arises from the valvular endocardium and its development in the cardiac chambers is extremely rare. A 52-year-old woman complained of palpitations and echocardiography revealed a cardiac tumor. Resection was performed via the right ventricle and main pulmonary artery under cardiopulmonary bypass. Histological examination of the resected tumor showed that it was a papillary fibroelastoma. The patient's postoperative course was unremarkable and no complications have been detected on followup.

## 1. Introduction

Primary cardiac tumors are rare and are usually benign. Cardiac myxoma is the most common tumor, while papillary fibroelastoma (PFE) is the second most common benign cardiac tumor and usually arises from the heart valves. It has been reported to account for approximately 14% of all primary cardiac tumors [1]. PFE can cause thromboembolism or mechanical interference with valvular function, so surgical therapy is indicated when the patient has symptoms or the tumor is mobile. We present a rare case of PFE arising from the free wall of the right ventricle.

## 2. Case Report

**Figure 1:**
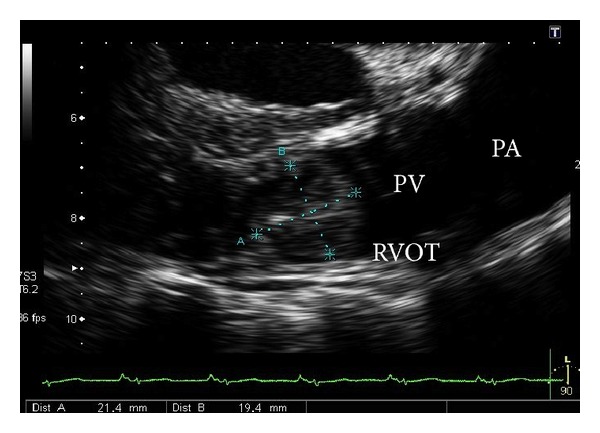
Transesophageal echocardiography shows the tumor in the right ventricular outflow tract. PA: pulmonary artery, RVOT: right ventricular outflow tract, and PV: pulmonary valve.

**Figure 2:**
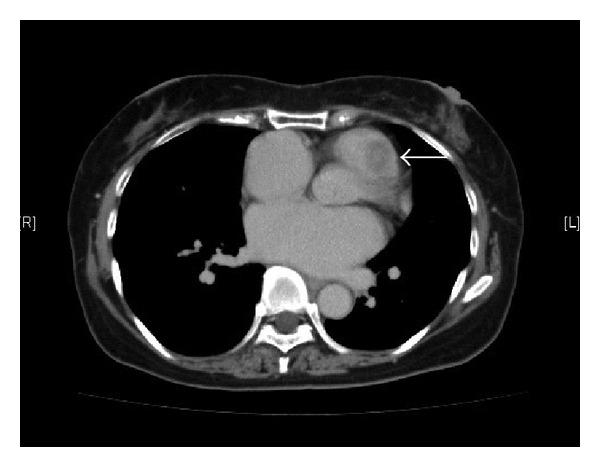
Chest computed tomography displays the tumor (white arrow) in the right ventricular outflow tract.

A 52-year-old woman was referred to our institution because of palpitations. She had no medical history. The electrocardiogram showed normal sinus rhythm, but echocardiography revealed a cardiac tumor. Transthoracic echocardiography (TTE) and transesophageal echocardiography (TEE) showed a mobile mass in the right ventricular outflow tract (Figure 1). Contrast-enhanced computed tomography also displayed a round mass in the right ventricle (Figure 2). On coronary angiography, the patient had normal coronary arteries and a feeding vessel for the tumor was not identified.

**Figure 3:**
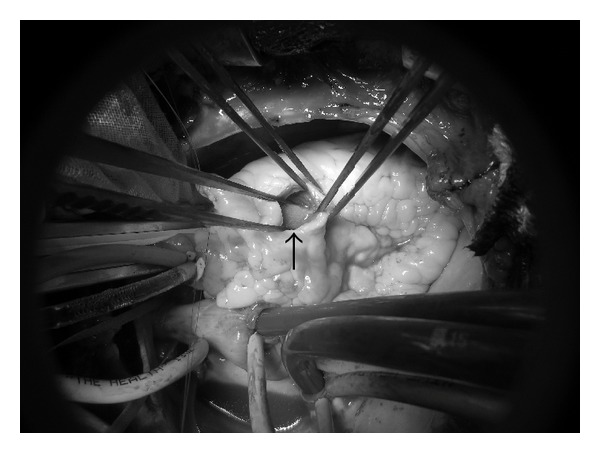
The tumor (black arrow) can be seen through an incision in the right ventricular free wall.

Surgical resection was performed via median sternotomy. Cardiopulmonary bypass was established from the superior and inferior vena cava to the ascending aorta. After cross-clamping the ascending aorta, cold crystalloid cardioplegic solution was administered antegradely via the aortic root. The main pulmonary artery was opened and the tumor was confirmed in the right ventricle via the pulmonary valve. The tumor was yellow-white and soft and was attached to the right ventricular free wall by a short stalk. Although the neck of the stalk could not be visualized, pulling downward on the tumor caused dimpling of the right ventricular free wall. After a small incision was made above the dimple, it was possible to identify the tumor stalk and the lesion could be resected along with the adjacent myocardium (Figure 3). Then the pulmonary artery and right ventricle were closed directly and the patient was weaned from cardiopulmonary bypass without any problems.

The excised tumor measured 22 × 18 mm. It was soft and yellowish, with a short stalk attached to the resected ventricular myocardium. Histopathological examination revealed that the tumor was a PFE that had been removed together with ventricular myocardium.

The postoperative course was unremarkable, and the patient has remained healthy during followup.

## 3. Discussion

PFE typically arises from the valvular endocardium and it most often affects the aortic valve (52%), followed by the mitral valve (16%), tricuspid valve (6%), and pulmonary valve (2%). PFE has also been reported in all cardiac chambers (28%), but such tumors are less frequent than those arising from the valves and it is extremely rare for the right side of the heart to be affected. The right ventricle was affected in 3.4% [2].

Although it is a benign tumor, PFE is associated with a high risk of ischemic or embolic complications and other fatal complications, especially when the mass arises in the left heart [3, 4]. When PFE arises in the right heart, the tumor may remain asymptomatic until it is large enough to interfere with hemodynamics. However, pulmonary embolism due to PFE of the right heart has been reported [5]. Pulmonary embolism can result in critical hypoxemia and pulmonary hypertension. Paradoxical embolic stroke has also been reported previously [6]. Therefore, a mobile intracardiac PFE or symptomatic tumor should be considered an indication for urgent resection. On the other hand, patients with asymptomatic nonmobile tumors can be followed up carefully by periodic clinical evaluation and echocardiography, undergoing surgical intervention when symptoms develop or the tumor becomes mobile [4].

In case of the right heart tumor, surgical resection can be achieved under the beating heart or cardiac arrest, with cardiopulmonary bypass using bicaval venous arterial cannulation. We considered that under the cardioplegic arrest was suitable for resection of the stalk with ventricular muscle.

PFE is an avascular tumor that contains a few fibroblasts and collagen and elastic fibers covered by hyperplastic endothelial cells [7]. In the present case also, a feeding artery was not detected by coronary angiography. The etiology of PFE is still controversial, previously have been reported the possibility of iatrogenic factors, organized thrombus, hamartomas, and true neoplasms [7].

In conclusion, we described a rare case of papillary fibroelastoma attached to the right ventricular free wall.
